# Mortality risk of chronic kidney disease: A comparison between the adult populations in urban China and the United States

**DOI:** 10.1371/journal.pone.0193734

**Published:** 2018-03-15

**Authors:** Jinwei Wang, Fang Wang, Rajiv Saran, Zhi He, Ming-Hui Zhao, Yi Li, Luxia Zhang, Jennifer Bragg-Gresham

**Affiliations:** 1 Renal Division, Department of Medicine, Peking University First Hospital; Institute of Nephrology, Peking University, Beijing, China; 2 Division of Nephrology, Department of Internal Medicine and the Kidney Epidemiology and Cost Center, University of Michigan, Ann Arbor, United States of America; 3 Department of Biostatistics and the Kidney Epidemiology and Cost Center, University of Michigan, Ann Arbor, United States of America; University of Utah School of Medicine, UNITED STATES

## Abstract

**Background:**

Chronic kidney disease (CKD) is a risk factor for all-cause mortality in the United States, but the evidence from China is limited. We investigate whether prognosis of CKD (mortality) differs between the two countries. In particular, we sought to compare the strength of association between CKD stage and all-cause mortality, by country.

**Methods:**

Mortality-linked data from China National Survey of Chronic Kidney Disease (urban population, n = 25,269) and US NHANES (2005–2010, n = 15,209) for adults >20 years old were analyzed. The Chinese cohort was followed until Dec 31, 2013, while the NHANES cohort until Dec 31, 2011. CKD was defined by eGFR <60ml/min/1.73m^2^ or albuminuria (defined as ACR ≥30mg/g). Weighted Cox models were used to evaluate the association between the two CKD indicators and mortality. Both stratified and combined models (with country interactions) were explored.

**Results:**

The Chinese sample had a lower proportion of eGFR<60 ml/min/1.73m^2^ (3.7% vs. 6.9%) and albuminuria (7.6% vs. 9.0%), compared to the US. Higher rates of mortality were observed with higher stages of CKD in both countries. HRs for mortality in the more advanced CKD categories reached 2.18 (1.14–4.15) in China and 1.66 (1.18–2.32) in the US in the absence of albuminuria, and 2.30 (1.13–4.68) and 3.04 (2.33–3.96) in the presence of albuminuria. No significant interactions were detected between country and these categories.

**Conclusion:**

The association between albuminuria and reduced eGFR and all-cause mortality was similar in both countries, with albuminuria being associated with the larger effect size compared to lower eGFR.

## Introduction

Chronic kidney disease (CKD) is an important public health problem around the world, with mean global prevalence of CKD (stages 1–5) of 13.4% based on a recent meta-analysis of nearly 100 published studies from around the world[1]. The 2012 Kidney Disease: Improving Global Outcomes (KDIGO) guideline defined CKD based on persistent reduced glomerular filtration rate (GFR) and/or albuminuria[2]. KDIGO supported the CKD Prognosis Consortium to investigate the relationship between kidney damage indicators and CKD prognosis, including progression to kidney failure, cardiovascular events and mortality. Based on 21 general population cohorts from around the world, Matsushita et al reported that both estimated GFR (eGFR) less than 60 ml/min/1.73m^2^ and urine albumin creatinine ratio greater than 10mg/g were independent predictors for all-cause mortality and cardiovascular specific mortality[3]. However, in this comprehensive meta-analysis, the data from Chinese population was from a local cohort with small sample size[4]. At the same time, the data of the US national representative population, from the National Health and Nutrition Examination Survey III (NHANESIII), was conducted nearly 20–30 years ago (1988–1994)[5].

China has experienced more than three decades of rapid economic growth accompanied by significant lifestyle changes[6]. A recent national survey has shown that CKD prevalence among adult Chinese population was 10.8%, with most patients in early stages, denoted by mildly reduced eGFR and presence of albuminuria[7]. However, in the US, Stage 3 CKD or worse is more common among CKD patients[8, 9]. Furthermore, based on the national database of in-patient Chinese population, Zhang et al found the main causes of CKD have shifted from glomerulonephritis related CKD in prior years to diabetes related CKD more recently. The crossing-over in underlying etiology of CKD occurred around 2010 and the trend has continued[10]. Hence, it can be foreseen that the etiology of CKD in China will be more and more similar to western countries with a diabetic nephropathy as the major etiology, given that risk factors for diabetes are shared by rapidly developing and developed nations across race/ethnicity[11, 12].

We hypothesized that, given the difference between China and the US in terms of ongoing ‘epidemiologic transitions’ in the former and previously reported differences with respect to prevalence of CKD by stage, that the relationship between mortality and CKD may be different between the two countries. Therefore, we sought to investigate the relationship between reduced eGFR and increased albumin to creatinine ratio (ACR) with the risk of mortality using nationally representative population-based samples from China and the US.

## Materials and methods

### Study population and outcomes ascertainment

Datasets from cross-sectional surveys within both China and the US were analyzed. For the US, data from the National Health and Nutrition Examination Survey (NHANES) were used[13]. The 2005–2006, 2007–2008 and 2009–2010 phases of NHANES, comparable to the study period of the Chinese survey were chosen for analysis. The NHANES data were publicly available, the analysts do not have access to information that could identify individual participants through the data analysis. The mortality-linked NHANES datasets were analyzed, which provided vital status for adult participants from the date of survey participation to December 31, 2011. The death information is derived from multiple sources, including the National Death Index (primary source), social security administration, as well as active follow-up. Under-reporting of death in NHANES is unlikely.

For China, the participants come from the China National Survey of CKD. The detailed description of the study design and procedure has been published previously[7]. Briefly, the survey used a multistage, stratified sampling method to get a representative non-institutionalized population of China (18 years and above). A total of 50,550 people were invited to participate, of whom 47,204 completed all essential investigations. All on-site investigations were done from January, 2007 through December, 2010. Then, the survey data were linked to the Master Death file of China Cause of Death Reporting System to determine all-cause mortality until December 31, 2013[14]. Personal IDs were used as the key variable for linking the data, and information including name, gender, birthdate, and home address was used for verifying the accuracy of linkage. Due to the potential significant incompleteness of death cases reporting in rural areas of China aroused by remarkably less number of deaths captured in hospitals than that in urban cities, we excluded participants from rural areas in the China National Survey of CKD. The under-reporting rate in urban areas was estimated to be 16.08% estimated during 2006–2008[15]. The China National Survey of CKD and the follow-up of participants were approved by the institutional review board (IRB) of Peking University First Hospital. The written informed consents have been obtained from all participants. Information that could identify individual participants were tightly kept secret, de-identified data were used through every step of data analysis.

Participants with missing data on serum creatinine or urine albuminuria, those less than 20 years of age, or pregnant, were excluded. Altogether, 25,269 individuals from urban areas of China and 15,209 individuals from the US were included in the final analysis. The comparison study protocol was approved by the ethics committee/IRB of Peking University Health Science Center.

### Indicators of chronic kidney disease

eGFR and ACR were used as the indicators of CKD. Serum creatinine was measured by the kinetic rate Jaffe method in both NHANES and China National Survey of CKD. The creatinine measurement in NHANES was recalibrated to standardized creatinine measurement obtained at the Cleveland Clinic Research Laboratory (Cleveland, Ohio)[16]. In the China National Survey of CKD, serum creatinine measurement was assayed in the central laboratory in each enrolled province. The calibration was traceable to an isotope dilution mass spectrometry reference method by using samples from the laboratory of Peking University First Hospital (Beijing, China). The GFR in both countries was estimated using the Chronic Kidney Disease Epidemiology Collaboration equation[17]. eGFR is categorized into three groups of ≥60ml/min/1.73m^2^, 45-59ml/min/1.73m^2^, and <45 ml/min/1.73m^2^, per the Kidney Disease Improving Global Outcomes-CKD definition/classification[2].

For NHANES 2005–2006, 2007–2008 and 2009–2010, random spot urine samples were obtained for participants at the mobile examination center. A solid-phase fluorescent immunoassay was used for the measurement of urine albumin level. Urinary creatinine was measured by modified kinetic Jaffe method using a Roche/Hitachi Modular P chemistry analyzer. For the China National Survey of CKD, a fresh morning spot urine sample or morning urine sample stored at 4°C for less than 1 week was used to measure urinary albumin and creatinine with the methods of immune-turbidimetric tests and kinetic Jaffe method, respectively. ACR (mg/g creatinine) was the ratio of the two measurements. We defined ACR of 30 mg/g or higher as albuminuria.

### Covariates

Covariates were selected based on their clinical relevance, as well as availability in both NHANES survey and China national survey of CKD. We used parallel definitions for each variable of the NHANES surveys and China National Survey of CKD. We categorized education into two levels as junior high school or lower (9 years of education or less) and high school or higher (9 or more years of education). Current smoking was defined as smoking every day for at least one year. Cardiovascular disease (myocardial infarction or stroke) was determined from diagnosis of doctors, which was self-reported by the participants. Blood pressure (BP) was measured by sphygmomanometer, three times at 1 min intervals. The mean of the three readings was calculated. Hypertension was defined as a systolic BP≥140 mmHg, or diastolic BP≥90 mm Hg, or self-reported use of antihypertensive medications in the last 2 weeks, or self-reported history of hypertension. Fasting blood glucose was measured enzymatically with a glucose oxidase method. Diabetes was defined as fasting plasma glucose≥126 mg/dl (7.1 mmol/L), or by the use of hypoglycemic agents, or any self-reported history of diabetes. Height and weight were measured according to the standard protocol. Body mass index (BMI) was calculated as weight in kilograms divided by height in meters squared.

### Statistical analysis

Continuous data are presented as mean (standard error) (SE), except for ACR, which were presented as median (interquartile range) because of the large skewness. The relevant characteristics were described by country. The follow-up time for each participant in the study was the length of time between the date of on-site examination and the end of follow up or date of death, whichever came first. In order to consider the complex sampling design for both the NHANES surveys and Chinese survey, the descriptive, survival, and regression analyses in separate dataset for each country were performed incorporating sampling weights. However, in the analyses based on the combined dataset for both countries, sampling weights were not used because of the lack of strata and cluster weight units in the Chinese dataset. All-cause mortality was calculated as the number of death per 1000 person-years. We calculated the mortality according to the eGFR categories and albuminuria status separately, as well as in each category of the combined eGFR and albuminuria levels.

Weighted Cox proportional-hazards models were employed separately in each country to evaluate the effect of the kidney damage indicators on mortality. Linear spline variables were introduced for both eGFR and ACR, with knots of eGFR at 15, 30, 45, 60, 90 (reference point) and 105 ml/min/1.73m^2^ and knots of ACR at 2, 5(reference point), 10, 30, 300 and 1000 mg/g. Multivariable adjusted hazard ratios (HR) and 95% confidence intervals (CI) are reported. Covariates included in the multivariable adjusted regression model were age (a continuous variable), gender (male vs. female), education (≥high school vs. <high school), current smoker (yes vs. no), cardiovascular disease history (yes vs. no), hypertension (yes vs. no), diabetes mellitus (yes vs. no) and BMI (a continuous variable). Proportional hazards assumptions were verified by testing the interaction with time using the likelihood ratio test, which yielded non-significant *P* values. We tested the linear spline association of eGFR and ACR with mortality, as well as the association of eGFR with mortality stratified by albuminuria status.

A combined dataset from both countries was created and multivariable Cox proportional-hazards models were used to evaluate the effect of each risk factor on mortality. Interaction terms between each of the risk factors and country were included in the model to evaluate differences of effect size by country. The combined categorical variable for eGFR and albuminuria levels was included in the model, with the status of eGFR≥60 ml/minute per 1.73m^2^ and no albuminuria used as the reference. Since participants in the 2009–2010 phase of NHANES have a limited period in the follow-up for mortality and may compromise the effect of CKD, a sensitivity analysis was done including only participants in the 2005–2006 and 2007–2008 phases.

All analyses were conducted by SAS software (version 9.4, SAS institute, CA, USA), except for linear spline analysis, which was conducted by Stata software (version 14.2, StataCorp LP, TX, USA). A *P* value of less than 0.05 was considered statistically significant.

## Results

The weighted means or proportions of demographic and clinical characteristics by country are shown in Table 1. The Chinese urban population was slightly older than the US population (mean age of 47.1 years versus 46.8 years), and had an equivalent proportion of males (48.0% versus 48.2%). However, the Chinese population had a lower proportion of high school education and above (66.9% versus 81.3%), as well as CVD (1.5% versus 5.4%), hypertension (31.6% versus 35.0%) and diabetes (7.1% versus 10.4%) compared with the US population. Meanwhile, the US population had a higher level of BMI than their Chinese counterparts. The level of eGFR was lower in the Chinese population than the US population, while the level of ACR was comparable between the two countries. The Chinese population had lower proportion of low eGFR and a slightly lower proportion of albuminuria than the US population.

**Table 1:** Comparison of Demographic Characteristics between China and the US*.

Measure	China	US
	(n = 25,269)	(n = 15,209)
Age (years)	47.1 (0.20)	46.8 (0.15)
Male (%)	48	48.2
High school or above (%)	66.9	81.3
Current smoker (%)	24.5	22.3
History of CVD (%)	1.5	5.4
Hypertension (%)	31.6	35.0
Diabetes (%)	7.1	10.4
Body mass index (kg/m^2^)	23.5 (0.04)	28.6 (0.07)
Creatinine (μmol/L)	0.87 (0.003)	0.89 (0.002)
eGFR (ml/min per 1.73m^2^)	91.8 (0.23)	93.6 (0.20)
low eGFR	3.7	6.9
ACR (mg/g)	6.3 (1.0–51.2)	6.3 (4.2–11.4)
Microalbuminuria (%)	7.1	7.7
Macroalbuminuria (%)	0.45	1.4
Any Albuminuria (%)	7.6	9.0
Low eGFR or albuminuria (%)	10.7	13.9

*Data are presented as Mean (SE) or %, except ACR is described as median (interquartile range). Sampling weights are applied to both cohorts. CVD = Cardiovascular disease; eGFR = Estimated glomerular filtration rate; ACR = Albumin creatinine ratio.

The median follow-up duration for the Chinese population was 6.9(IQR: 5.0–7.6) years, and that for the US population was 3.7 (IQR: 2.3–5.2) years. The US population had a significantly higher mortality rate than the Chinese population. Both US and Chinese population showed higher mortality rate at lower levels of eGFR, presence of albuminuria and the combination of lower eGFR and albuminuria (Table 2 and Table 3).

**Table 2:** Crude Death Rates of the participants from China.

Measure	No Albuminuria			Albuminuria			Total		
	Number	Number of Events	Events/1000 person-yrs*	Number	Number of Events	Events/1000 person-yrs*	Number	Number of Events	Events/1000 person-yrs*
eGFR ≥ 60	22,466	362	2.7	1663	51	5.0	24,129	413	2.9
45 ≤ eGFR < 60	811	31	10.4	152	10	29.6	963	41	13.3
eGFR < 45	99	10	19.5	78	8	48.8	177	18	29.0
Total	23,376	403	3.0	1893	69	7.0	25,269	472	3.3

Note

*means weighted.

**Table 3:** Crude Death Rates of the participants from the US.

Measure	No Albuminuria			Albuminuria			Total		
	Number	Number of Events	Events/1000 person-yrs*	Number	Number of Events	Events/1000 person-yrs*	Number	Number of Events	Events/1000 person-yrs*
eGFR ≥ 60	12,417	299	4.4	1369	119	17.1	13,786	418	5.4
45 ≤ eGFR < 60	699	70	19.6	227	58	69.5	926	128	28.7
eGFR < 45	239	43	44.3	258	84	114.0	497	127	74.5
Total	13,355	412	5.5	1854	261	31.2	15,209	673	7.8

Note

*means weighted.

**Fig 1:**
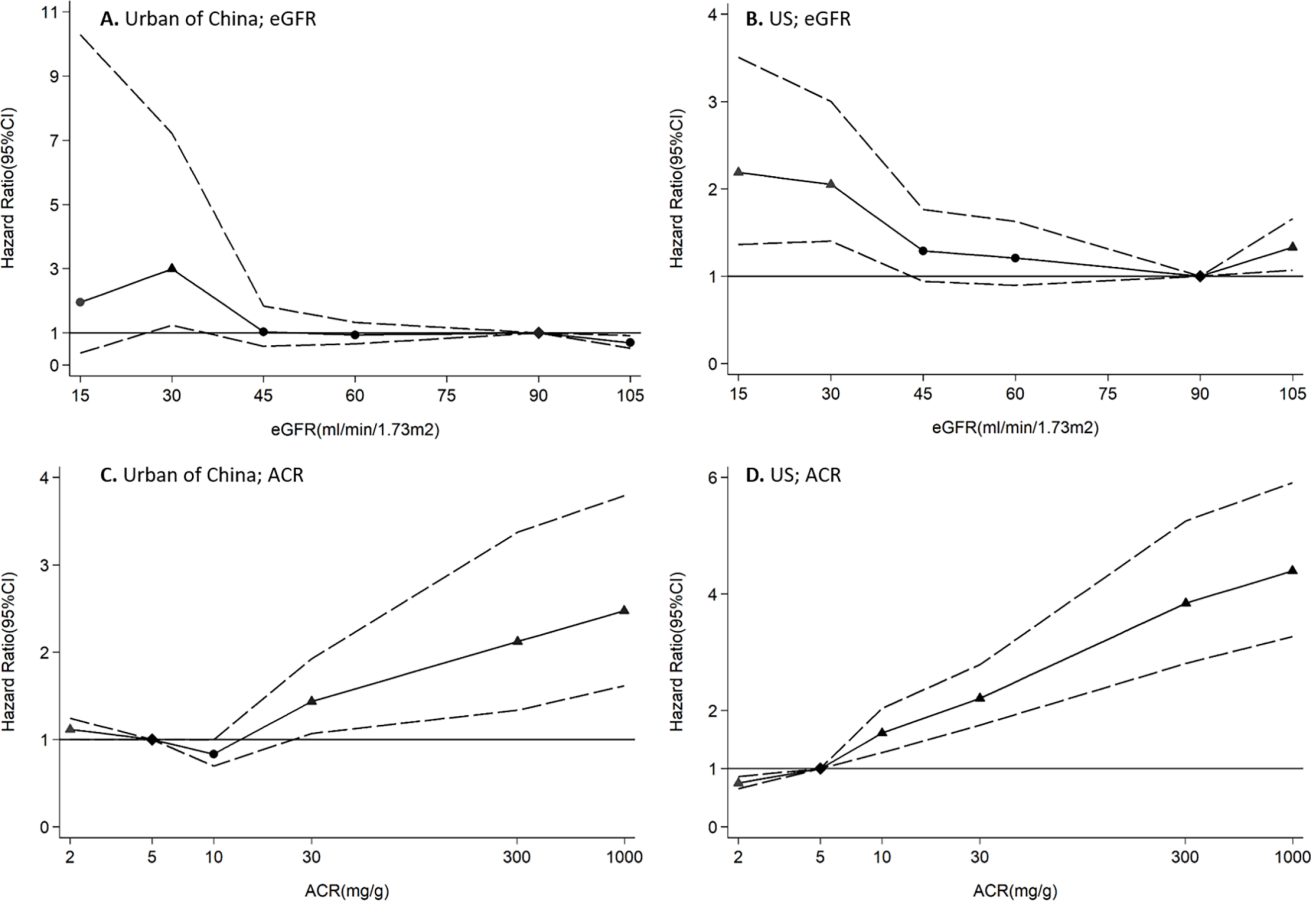
HRs and 95% CIs for all-cause mortality according to spline eGFR and ACR in urban China and the US. HRs and 95% CIs (area between dashed lines) according to eGFR and ACR in China (A, C) and US (B, D). The associations were adjusted for age, sex, education level, smoking, history of cardiovascular disease, hypertension, diabetes, body mass index, and ln(ACR)(when testing for spline eGFR)/eGFR(when testing for spline ACR). The reference (diamond) was eGFR 90 mL/min/1·73 m^2^ and ACR 5 mg/g, respectively. Triangles represent statistically significant and circles represent not significant. Abbreviation: HR = Hazard ratio, CI = Confidence interval, eGFR = Estimated glomerular filtration rate, ACR = Albumin-to-creatinine ratio.

**Fig 2:**
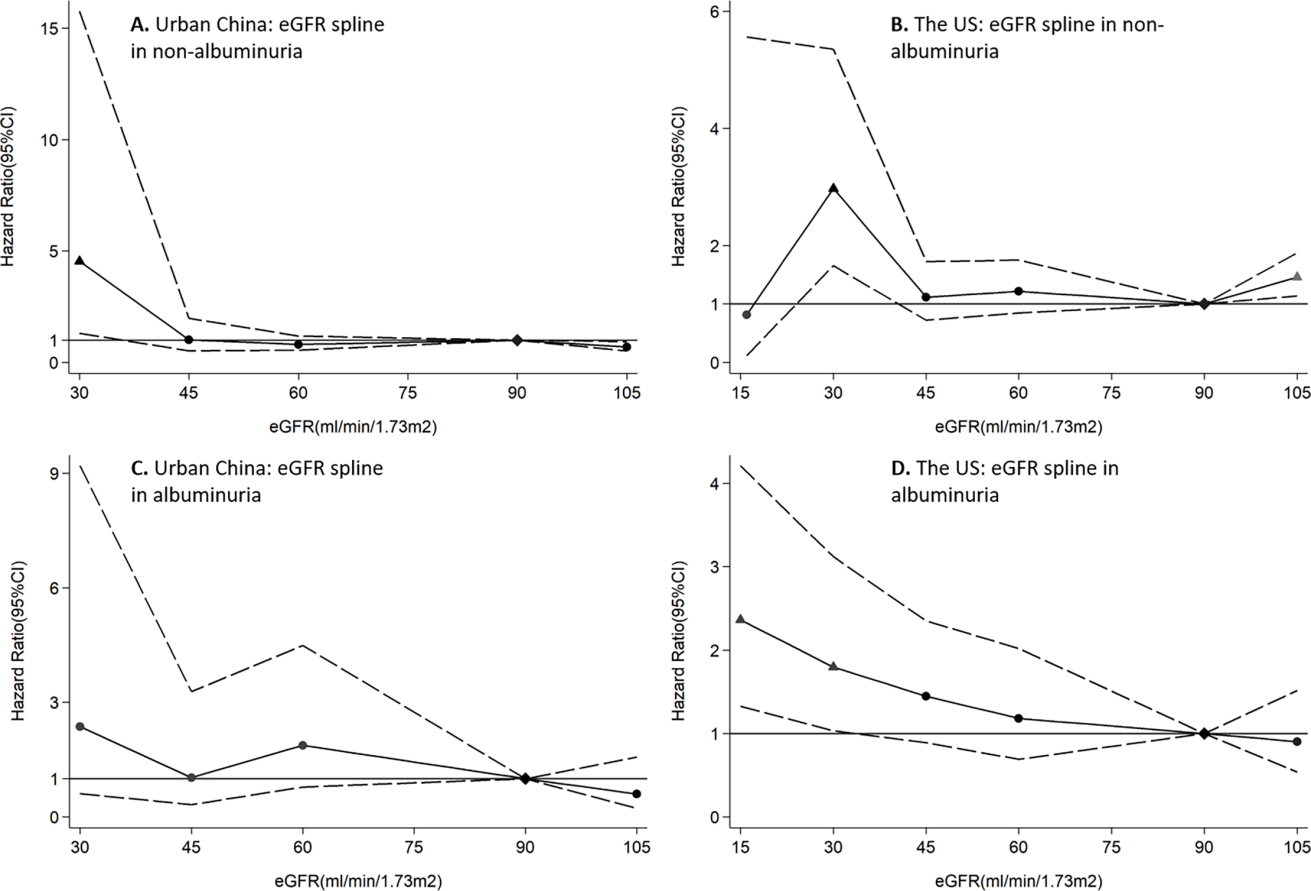
HRs and 95% CIs for all-cause mortality according to spline eGFR stratified by presence of albuminuria in urban China and the US. HRs and 95% CIs (area between dashed lines) according to eGFR in China (A, C) and US (B, D). The associations were adjusted for age, sex, education level, smoking, history of cardiovascular disease, hypertension, diabetes, body mass index, and ln(ACR). The reference (diamond) was eGFR 90 mL/min/1·73 m^2^. Triangles represent statistically significant and circles represent not significant. Abbreviation: HR = Hazard ratio, CI = Confidence interval, eGFR = Estimated glomerular filtration rate, ACR = Albumin-to-creatinine ratio.

In spline analysis by country, eGFR of ≤30 ml/min/1.73m^2^ was significantly associated with higher risk of mortality in the US. A higher risk of mortality was observed with higher ACR in both countries, while the significant association first emerged at ACR of 30mg/g in the Chinese population and at 10mg/g in the US population (Fig 1). When the splined eGFR were stratified by albuminuria, we can observe among the US population that declined eGFR showed increased risk of mortality when at levels of ≤30 ml/min/1.73m^2^ when albuminuria is present, while the corresponding associations were not significant at 15 ml/min/1.73m^2^ of eGFR when albuminuria is not present. Due to the remarkably wide confidence interval in effect measurement, HRs at 15 ml/min/1.73m^2^ of eGFR were not shown in the Chinese population. Significant associations can be observed at 30 ml/min/1.73m^2^ of eGFR when albuminuria is present, but is null when albuminuria is absent (Fig 2).

In the Cox regression analyses based on the combined dataset from both countries, we observed a trend of increased risk for mortality through the decreased levels of eGFR in both the presence and absence of albuminuria. The significant associations can be detected when eGFR was less than 45 ml/min/1.73m^2^ when albuminuria was absent among both the Chinese and US population. When albuminuria was present, the significant can be observed through all eGFR levels in both countries. No significant interactions were detected between each level of the combined kidney damage indicator and country (Table 4). Consistent results were found in the sensitivity analysis after excluding the 2009–2010 NHANES data (n = 5,611) (S1 Table.).

**Table 4:** Hazard ratios for all-cause mortality with interactions by risk factors in the combined dataset.

Measure	China			The US		*P* for interaction with country
	HR	95%CI		HR	95%CI	
Age (per 1 year)	1.05	1.04–1.06		1.08	1.07–1.09	<0.001
Male	1.54	1.25–1.89		1.38	1.17–1.62	0.42
High school and above	0.77	0.63–0.94		0.81	0.69–0.95	0.71
Smoking	1.24	0.99–1.57		1.85	1.48–2.30	0.02
CVD	1.47	1.07–2.02		1.53	1.28–1.84	0.82
Hypertension	1.34	1.09–1.65		1.07	0.90–1.27	0.10
Diabetes	1.25	0.98–1.60		1.36	1.14–1.62	0.60
Body mass index (kg/m^2^)	0.96	0.93–0.98		0.97	0.96–0.99	0.28
No Albuminuria						
eGFR ≥ 60	1.00	Reference		1.00	Reference	
45 ≤ eGFR < 60	1.05	0.72–1.54		1.03	0.78–1.36	0.93
eGFR < 45	2.18	1.14–4.15		1.66	1.18–2.32	0.46
Albuminuria						
eGFR ≥ 60	1.54	1.14–2.08		1.77	1.42–2.21	0.47
45 ≤ eGFR < 60	1.71	0.90–3.24		2.36	1.75–3.19	0.37
eGFR < 45	2.30	1.13–4.68		3.04	2.33–3.96	0.47

Note: HR = Hazard ratio; CI = Confidence interval; CVD = Cardiovascular disease; eGFR = Estimated glomerular filtration rate.

## Discussion

All-cause mortality rates were shown to be higher among lower eGFR categories, in the presence of albuminuria, and in the combined categories of the two indicators among general non-institutionalized adults in urban China and the US in present study. The association of lower eGFR and higher ACR with all-cause mortality were detected in both two countries. Additionally, reduced eGFR was shown to be associated with a higher risk of all-cause mortality in the presence of albuminuria than absence of albuminuria. However, no interactions between kidney damage indicators and country emerged in the analysis using the combined dataset.

In this study, nationally representative population samples from the US and a representative urban population in China were used in making comparisons of mortality as an outcome among those with CKD. Although the US population had an obviously shorter follow-up term than the Chinese population, a mortality rate of more than double was observed. While these findings may be due to higher prevalence of comorbidities and obesity in the US population, it could also result from under reporting of deaths in China[15]. However, the direction of the association between low eGFR and/or the presence of albuminuria and higher all-cause mortality, was consistently observed in both countries.

The association of reduced eGFR and increased ACR with all-cause mortality among general population has been established based on results of comprehensive meta-analysis[3, 18]. Our study’s findings from analysis of recent samples from US and China are consistent with those prior publications. We also tested the association between eGFR and ACR and all-cause mortality in a paper including both rural and urban data of Chinese. The results from the prior publication were consistent with the current study but own a wider CI of HR due to the limited study power[19]. When eGFR and ACR were treated as continuous variables in our study, the patterns of the association were similar between the two countries. However, the association between linear spline eGFR and mortality appeared clearer in the US population compared with the Chinese population below eGFR of 45ml/min/1.73m^2^. This association was statistically non-significant in the Chinese sample, potentially due to the limited number of death events and or sample size at low eGFR. A similar pattern between the two countries was observed with respect to ACR. The higher risk of mortality was observable from 10mg/g of ACR in the US, and 30mg/g in China. Given similar patterns for these associations in both countries, the absence of an interaction between CKD (using both indicators) and country was reasonable in the combined dataset. This finding was consistent with a previous study, which demonstrated that the magnitude of effect of eGFR and ACR on mortality was similar among races[20].

The association of albuminuria on risk of mortality was important in both countries. In the combined dataset, among Chinese people with no albuminuria, a significant risk of mortality could be detected only in the subgroup with eGFR<45 ml/min/1.73m^2^, whilst it could be detected among individuals with eGFR>60 ml/min/1.73m^2^ when albuminuria was present. Similarly, significant association with mortality could be identified in the subgroup with eGFR<45 ml/min/1.73m^2^ in the absence of albuminuria in the US sample, while graded risks were detected through eGFR levels when albuminuria was present. Albuminuria is considered an early marker of kidney damage. A recent meta-analysis, including 637,315 individuals without a history of cardiovascular disease, demonstrated that ACR can be a strong predictive marker for cardiovascular mortality compared to eGFR, based on the c-statistic difference and reclassification improvement in addition to the traditional risk factors[21]. However, in the management of CKD, albuminuria may be underemphasized relative to reduced kidney function in routine clinical practice. In the KDIGO-CKD guideline, definition of stage1 and stage2 CKD incorporates a component of albuminuria, however, in advanced stages; the definition is only based on the level of reduced eGFR[2]. Clearly, quantification of albuminuria/proteinuria deserves more attention placed in the management of CKD, irrespective of country.

Although the prevalence of CKD is almost equivalent between China and the US when considering the combined measure of low eGFR and increase ACR, the proportion of the two CKD indicators was different. With respect to CKD in China there were twice as many people with albuminuria than those with low eGFR, while in the US, the prevalence difference of low eGFR and albuminuria was much smaller than in China. Wen et al reported the prevalence of CKD and its stages among general population in Taiwan[22], where the ethnicity and living habits were the same with in mainland China, but the economic development was 10–20 years ahead, and found a higher proportion of lower eGFR (CKD stage 3 or worse) than that reported among population in mainland China[7]. Thus, it can be hypothesized that the proportion of individuals with reduced eGFR will increase in mainland China, if effective measures were to be taken to curb the epidemic of CKD. Besides the composition of CKD indicators, a recent study based on hospitalized CKD patients around China also suggested the potential change of etiology of CKD from glomerulonephritis related CKD to diabetes mellitus related CKD[10]. Given the above facts, as well as the findings of similar pattern for mortality risk from kidney damage between China and the US, actions should be taken in both China and the US, to deal with the epidemic of CKD to prevent pre-mature deaths.

Although our study has advantages of using national representative populations in both China and the US, some limitations should be recognized. First, the laboratory assay and calibration methods have some differences for the measurement of creatinine and albumin between China and US and may lead to certain inconsistency; Second, no repeated measure for the creatinine and albumin were conducted, and thus the ‘persistence’ criterion of reduced kidney function or presence of albuminuria to define CKD may not be satisfied, which could lead to slight overestimation of CKD prevalence. Finally, as discussed, incomplete ascertainment of death could be occurring in China and could potentially lead to the underestimation of mortality risk in that country.

## Conclusion

Our study made a comprehensive comparison of mortality associations with eGFR and albuminuria between urban China and the US. Although the two CKD indicators showed more obvious risk for mortality in the US than in urban China, no significant interaction by country was found. We believe that while opportunities for prevention of kidney disease are significant in both countries, the higher prevalence of earlier stages of the disease in that country should serve as an imperative for aggressive primary and secondary preventive efforts to preempt the devastating and costly downstream consequences of CKD.

## Supporting information

S1 Table Hazard ratios for all-cause mortality with interactions by risk factors in the combined dataset with only 2005–2006 and 2007–2008 phases of NHANES included. Note: NHANES = the National Health and Nutrition Examination Survey; HR = Hazard ratio; CI = Confidence interval; CVD = Cardiovascular disease; eGFR = Estimated glomerular filtration rate.
(DOCX)
