# Supplementary material for: Mortality risk of chronic kidney disease: A comparison between the adult populations in urban China and the United States
Source: PLoS One. 2018 Mar 15;13(3):e0193734. doi: 10.1371/journal.pone.0193734 (PMC5854279; doi:10.1371/journal.pone.0193734)
Supplement: S1 Table — Note: NHANES = the National Health and Nutrition Examination Survey; HR = Hazard ratio; CI = Confidence interval; CVD = Cardiovascular disease; eGFR = Estimated glomerular filtration rate. (DOCX) [file pone.0193734.s001.docx]

| Measure | China | | |  | The US | | *P* for interaction with country |
| --- | --- | --- | --- | --- | --- | --- | --- |
|  | HR | 95%CI | |  | HR | 95%CI |  |
| Age (per 1 year) | 1.05 | 1.04-1.06 | |  | 1.08 | 1.08-1.09 | <0.001 |
| Male | 1.54 | 1.25-1.89 | |  | 1.30 | 1.09-1.55 | 0.23 |
| High school and above | 0.77 | 0.63-0.94 | |  | 0.81 | 0.68-0.95 | 0.75 |
| Smoking | 1.24 | 0.99-1.57 | |  | 1.96 | 1.54-2.49 | 0.008 |
| CVD | 1.47 | 1.07-2.02 | |  | 1.54 | 1.27-1.88 | 0.79 |
| Hypertension | 1.34 | 1.09-1.65 | |  | 1.04 | 0.86-1.25 | 0.07 |
| Diabetes | 1.25 | 0.98-1.60 | |  | 1.38 | 1.14-1.67 | 0.54 |
| Body mass index (kg/m^2^) | 0.96 | 0.93-0.98 | |  | 0.98 | 0.96-0.99 | 0.22 |
| No Albuminuria |  |  |  |  |  |  |  |
| eGFR ≥ 60 | 1.00 | Reference | |  | 1.00 | Reference |  |
| 45 ≤ eGFR < 60 | 1.05 | 0.72-1.54 | |  | 1.04 | 0.78-1.39 | 0.95 |
| eGFR < 45 | 2.18 | 1.14-4.15 | |  | 1.52 | 1.05-2.20 | 0.34 |
| Albuminuria |  |  | |  |  |  |  |
| eGFR ≥ 60 | 1.54 | 1.14-2.08 | |  | 1.72 | 1.35-2.18 | 0.59 |
| 45 ≤ eGFR < 60 | 1.71 | 0.90-3.24 | |  | 2.37 | 1.73-3.26 | 0.37 |
| eGFR < 45 | 2.30 | 1.13-4.68 | |  | 2.86 | 2.14-3.82 | 0.58 |
